# Variables Associated with Effects on Morbidity in Older Adults Following Disasters

**DOI:** 10.1371/currents.dis.0fe970aa16d51cde6a962b7a732e494a

**Published:** 2014-12-05

**Authors:** J Lee Jenkins, Matthew Levy, Lainie Rutkow, Adam Spira

**Affiliations:** Emergency Medicine, Johns Hopkins University School of Medicine, Baltimore, Maryland, USA; Emergency Medicine, Johns Hopkins University School of Medicine, Baltimore, Maryland, USA; Health Policy and Management, Johns Hopkins University School of Medicine, Baltimore, Maryland, USA; Department of Mental Health, Johns Hopkins University School of Medicine, Baltimore, Maryland, USA

## Abstract

Introduction: Older adults are vulnerable to disproportionately higher morbidity following disasters. Reasons for this vulnerability are multifaceted and vary by disaster type as well as patient comorbidities. Efforts to mitigate this increased morbidity require identification of at-risk older adults who can be targeted for intervention. Methods: A PubMed search was performed using the search terms “geriatric, disaster” and “morbidity, disaster” to identify published articles that reported variables associated with increased morbidity of older adults during and after disasters. A review of article titles and abstracts was then conducted to identify those articles that contained evidence-based variables that render older adults vulnerable to poor health outcomes during disasters. Results: A total of 233 studies was initially identified. After applying exclusion criteria, nine studies were chosen for the comprehensive review. Based on the synthesis of the literature, factors were identified that were repeatedly associated with morbidity and mortality among older adults during and shortly after disasters. Conclusion: Older adults, especially those with multiple co-morbidities, are at risk of increased morbidity after disasters and catastrophic events. Factors such as the need for prescription medications, low social support, visual and hearing impairment, impaired mobility, and poor economic status are associated with an increased risk of morbidity.

## Introduction

Older adults are especially vulnerable to disaster-related morbidity.^1^ However, the proportion of the older population at elevated risk for these outcomes remains unclear. Health agencies may be limited in their ability to identify at-risk elders, develop interventions to mitigate the impact of disasters on older adults’ well-being, and plan appropriate disaster response.

Vulnerability of Community-Residing Older Adults to Disasters

Across the United States (US), the average age of the population continues to increase, with the majority of older adults living in the community with varying levels of social support. 2 Community-dwelling older adults are at increased risk of morbidity from disasters and public health emergencies such as power outages, hurricanes, pandemics, and heat waves 3. Numerous studies demonstrate that disasters disproportionately affect older adults 4 ^,^ 5 ^,^ 6 ^,^ 7 ^,^ 8 ^,^ 9 ^,^ 10 ^,^ 11 .

For example, prior to Hurricanes Katrina and Rita in 2005, adults aged 60 or older made up only 15% of the population of New Orleans, Louisiana 12 ^,^ 13. However, 71% of those who died because of the hurricane were over age 65 14. During the 1995 heat wave in the Midwest, the median age of the 465 people in Chicago whose deaths were heat-related was 75 years of age 15. Older adults are at more of an increased risk from heat waves than almost any other disaster due to loss of power, loss of air conditioning, and exacerbation of chronic diseases 16 ^,^ 17 . These outcomes may have been mitigated if at-risk older adults had been targeted for intervention.

During a major disaster, a region’s medical resources may be overwhelmed. It is usually not possible for resources from out of state, such as disaster medical teams, to begin delivery of scene care within the first 72 hours of a disaster 18. It is therefore necessary for older adults, members of their social networks, and first responders to focus on managing needs that occur after the initial phase of the disaster response. These needs are diverse and include ensuring the availability of prescription medications, and delivery of meals and personal care items. Pharmacies are often closed or without power and older adults may not have transportation or the ability to obtain additional medications.

To identify factors associated with increased morbidity of older adults during and shortly after disasters, we reviewed published articles in this domain.

## Methods

The National Library of Medicine's MEDLINE database was searched utilizing the PubMed search engine. Specifically, the search terms “geriatric, disaster” and “morbidity, disaster” were used to identify published articles that reported variables associated with increased morbidity of older adults during and after disasters. A review of article titles and abstracts was then conducted between January to March 2014 to identify those articles that contained evidence-based variables that render older adults vulnerable to poor health outcomes during disasters.

Articles that studied a population aged 55 and older were included to capture a larger proportion of the literature. Articles were excluded for the following reasons: 1) no data pertaining to adults >55; 2) no data pertaining to a disaster; 3) review articles without original data; 4) studies of nursing home patients; and 5) no risk factors identified that were present prior to the disaster. Please refer to Figure 1 for further detail.

**Review Methodology:**
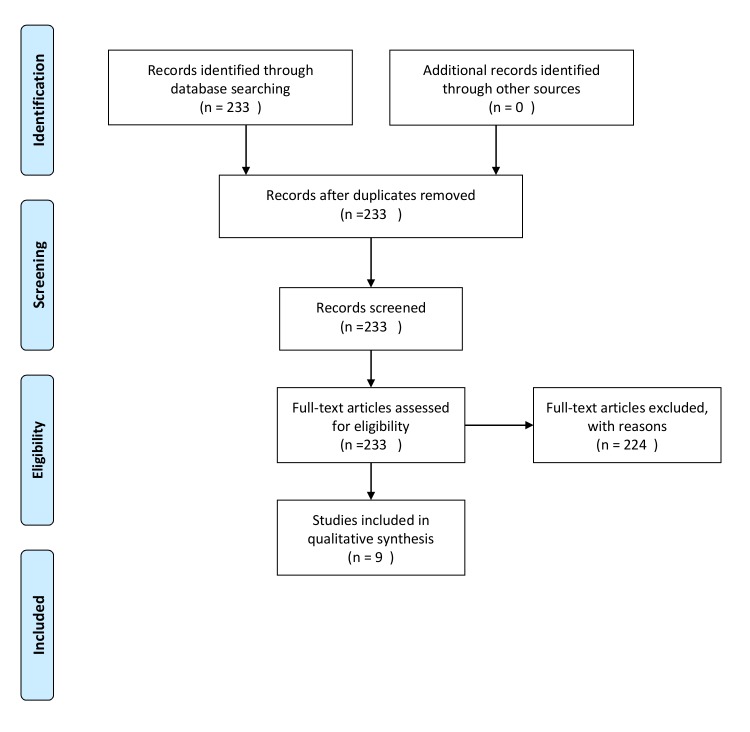

## Results

We initially identified 233 studies. After applying the exclusion criteria, nine studies were chosen for review. These included quantitative analyses, qualitative analyses, policy analyses, and expert observations from specific disasters. The references of each identified study were scanned to identify additional studies of interest. Based on the synthesis of the literature, we identified factors that were repeatedly associated with morbidity and mortality among older adults during and shortly after disasters.

Several studies specifically identified evidence-based vulnerability factors that render older adults vulnerable to poor health outcomes during disasters (Table 1). The most frequently cited risk factors for increased morbidity in community-residing older adults after a disaster were the presence of multiple co-morbidities^7^^,^ 16 ^,^ 19 ^,^ 20 ^,^ 21 and low social support 16 ^,^ 21 ^,^ 22 ^,^ 23 . Other risk factors mentioned in evidenced-based studies have included the need for multiple prescription medications 22, poor economic status 7, visual and hearing impairment 24, impaired mobility^24^ , pets in the home 20 , cognitive impairment 24 , being a racial minority 25, history of psychiatric illness^24^, religious beliefs 25, and female gender 25. Disaster types cited in evidence-based studies included heat waves, hurricanes, and earthquakes. Although exacerbation of chronic illnesses has been shown repeatedly to account for the majority of disaster-related morbidity 18, increased morbidity may also stem from decreased evacuation and increased acute injuries.

**Figure:**
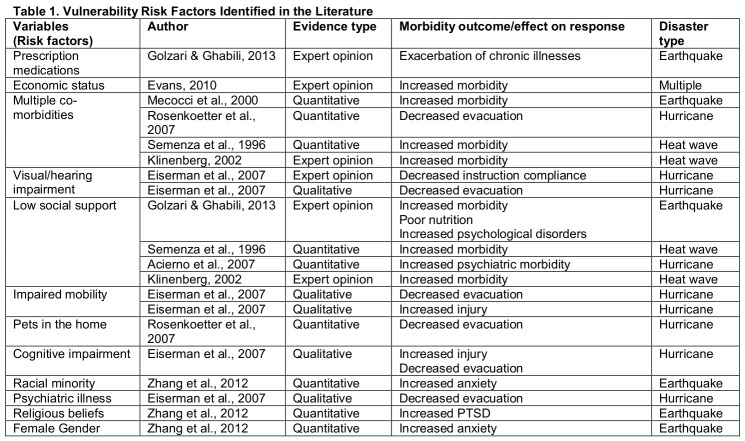


******

The top discharge diagnosis from one clinic in Louisiana after Hurricane Katrina was medication refills; other frequent visits were due to exacerbations of chronic diseases 18. In the same article, it was noted that medication refills were also the top reason for visits to another clinic in Mississippi after Hurricane Katrina. In another study, a significant portion of households reported unmet health care needs during the massive evacuations after the 2007 Southern California wildfires 28. The majority of adults taking prescription medications left them at home, and the request for replacement prescription medication was reported by the head of household in 29.2% of families. These articles describe the presence of a need for prescription medications and medical care after a disaster.

## Discussion

Older adults living in the community are a heterogeneous group with varying levels of physical and mental health needs, social and family support, living arrangements, and incomes 11. They also differ in their level of preparedness for and needs during a disaster or large-scale emergency 26. However, little is known about the prevalence of pre-existing disaster-related vulnerabilities among older adults across regions or the entire country, and existing studies often investigate only one catastrophic event.**To minimize the impact of disasters on older adults, it is essential to first conduct an accurate community-based assessment of older adults’ vulnerabilities and utilize this assessment for improved planning 27.

In addition to the general population of community-residing older adults in whom the prevalence of pre-existing vulnerabilities has not been determined, “frail” older adults, defined as those with serious, chronic health conditions such as heart disease, have not received significant consideration. Determining the prevalence of vulnerability amongst this population is especially important given that they are more likely than younger and healthier residents to need extra assistance with evacuations and recovery from disasters 8. In fact, at least 13 million adults aged 50 years or older in the United States have said they would need help to evacuate during a disaster, and about half of these would require help from someone outside their household 9.

The response of public safety officials and first responders would also be enhanced by the study of the prevalence of vulnerability factors. In addition, if these vulnerabilities were known beforehand, the same public safety personnel could work to mitigate these factors. Public safety officials, such as the local fire department and emergency medical services (EMS), can serve as a vehicle for outreach and resource to deliver these needs 26.

Disaster-Relevant Research Conducted in Advance of a Disaster

Much research in disaster medicine is performed during the disaster and in real-time situations, with few resources for planning or an evidence-based approach 3. These challenges for research and data collection during disasters and catastrophic events make planning for research even more important 29. The use of pre-established, community networks has been shown to improve coordinated disaster planning and response. However, these community networks, when in place, continue to rely upon needs assessments and vulnerability data after a disaster to assist them in these endeavors. By providing existing community response networks with results of disaster research and evidence-based tools for preparedness in advance of an event, public health agencies may strengthen their planning and response efforts and reduce morbidity.

Other experts in disaster medicine have also called for research to be conducted before disasters to inform evidence-based planning and response. Although disaster relief operations may be data driven, they are not necessarily evidence-based. This is especially the case when needs assessments are performed after disasters, yet no data exist regarding the prevalence of vulnerability factors. Options for strengthening evidence-based activities include rigorously adhering to evidence-based interventions, using evidence-based tools to identify new approaches to problems of concern, and improving standards for evidence of effectiveness in disaster science and services 30. In addition, research that focuses on the older adult population in disasters is uncommon and collecting data in post-disaster settings is fraught with technical and ethical difficulties 7.

Although not identified in the literature search as a vulnerability factor, it has been noted that electricity loss may put older adults at increased risk during disasters. Older adults are more vulnerable to hypothermia and hyperthermia during temperature extremes, and may suffer during energy loss in a climate controlled home. A loss of electrical power also prevents the use of needed medical equipment, such as nebulizers and home oxygen therapy 31. Recently, a preparedness drill in which we used to demonstrate how Medicare claims data may be used to identify individuals with electricity-dependent durable medical equipment during a disaster and securely disclosed it to a local health department. 32

Utilization of Existing Databases for Disaster Research

Previous research has identified characteristics of community-residing older adults that predispose them to increased morbidity during disasters 8.******However, determining the prevalence of their vulnerabilities remains a challenge.******As mentioned above, research conducted during disasters is methodologically challenging and often does not provide the data necessary for planning and response. Adequate planning and educational tools require information regarding the prevalence of risk factors. At a 2009 National Academies meeting, consensus was reached regarding the importance of mining existing data for insights and leveraging ongoing epidemiological and population surveys for pre-disaster baseline data 3. The use of existing databases efficiently harnesses the strengths of methodologically rigorous study designs and has the potential to build an interdisciplinary collaboration to solve these critical problems.

Through the utilization of pre-existing databases, it may be possible to identify vulnerabilities of older adults prior to disasters. Ideally, the databases would be large and detailed enough to identify by neighborhood or community, which localities have the greatest need for specific services.

**Conclusion**

Older adults are at a known risk for increased morbidity during and shortly after disasters and catastrophic events, especially in the presence of multiple co-morbidities such as hypertension, diabetes, stroke, and heart disease. Other factors, such as the need for prescription medications, low social support, visual and hearing impairment, impaired mobility, and poor economic status, have also been associated with an increased risk of negative health outcomes. The utilization of existing databases of older adults’ vulnerabilities and the measurement of the prevalence of these risk factors in the community may enhance disaster research and response methodologies and decrease disaster-related morbidities.
